# Leukoplakia: An Invasive Cancer Hidden within the Vocal Folds. A Multivariate Analysis of Risk Factors

**DOI:** 10.3389/fonc.2021.772255

**Published:** 2021-12-13

**Authors:** Hanna Klimza, Wioletta Pietruszewska, Oskar Rosiak, Joanna Morawska, Piotr Nogal, Małgorzata Wierzbicka

**Affiliations:** ^1^ Department of Otolaryngology and Laryngological Oncology, Poznań University of Medical Sciences, Poznań, Poland; ^2^ Department of Otolaryngology, Head and Neck Oncology, Medical University of Lodz, Lodz, Poland; ^3^ Balance Disorder Unit, Department of Otolaryngology, Medical University of Lodz, Lodz, Poland

**Keywords:** vocal fold leukoplakia, narrow band imaging (NBI), glottic cancer, white light, anterior commissure (AC)

## Abstract

**Introduction:**

Discerning the preoperative nature of vocal fold leukoplakia (VFL) with a substantial degree of certainty is fundamental, seeing that the histological diagnosis of VFL includes a wide spectrum of pathology and there is no consensus on an appropriate treatment strategy or frequency of surveillance. The goal of our study was to establish a clear schedule of the diagnostics and decision-making in which the timing and necessity of surgical intervention are crucial to not miss this cancer hidden underneath the white plaque.

**Material and Methods:**

We define a schedule as a combination of procedures (white light and Narrow Band Imaging diagnostic tools), methods of evaluating the results (a combination of multiple image classifications in white light and Narrow Band Imaging), and taking into account patient-related risk factors, precise lesion location, and morphology. A total number of 259 patients with 296 vocal folds affected by leukoplakia were enrolled in the study. All patients were assessed for three classifications, in detail according to Ni 2019 and ELS 2015 for Narrow Band Imaging and according to Chen 2019 for white light. In 41 of the 296 folds (13.9%), the VFL specimens in the final histology revealed invasive cancer. We compared the results from the classifications to the final histology results.

**Results:**

The results showed that the classifications and evaluations of the involvement of anterior commissure improve the clinical utility of these classifications and showed improved diagnostic performance. The AUC of this model was the highest (0.973) with the highest sensitivity, specificity, PPV, and NPV (90.2%, 89%, 56.9%, and 98.3%, respectively).

**Conclusion:**

The schedule that combines white light and Narrow Band Imaging, with a combination of the two classifications, improves the specificity and predictive value, especially of anterior commissure involvement.

## Introduction

Vocal fold leukoplakia (VFL) is a clinical term that describes a white patch or plaque resulting from epithelial parakeratosis, but does not specify what is hidden within the lesion. The histological diagnosis of VFL includes a wide spectrum of pathology, through stages of dysplasia to invasive cancer (1, 2). Additionally, there is no consensus on the threshold for surgical intervention, appropriate treatment strategy (wait and see policy, sampling, or excisional surgery), or the frequency of surveillance (3, 4). Therapeutic decisions balance high-quality voice preservation and oncological safety, thus caution is taken while referring a patient for surgical treatment and techniques are chosen to eliminate a superficial lesion while preserving the underlying vibratory mucosa. Some authors claim that a ‘wait and see’ approach is appropriate for smooth lesions, while some believe that more aggressive treatment should be performed (5, 6). In any event, the most popular treatment of VFL is surgery, even though in 50% of specimens, the final histology does not show dysplasia or cancer (7, 8). In aiming to resolve these discrepancies, diagnostic methods and recommendations such as the schedule for VFL management should be of utmost importance (9).

White light (WL) laryngoscopy is the first line and the most important diagnostic tool in the assessment of the pathologies of the larynx, including VFL (10, 11). However, it has limitations, especially in the assessment of underlying vascularization, which can be resolved by using additional light settings. Narrow Band Imaging (NBI) is a well-established bioendoscopic technique that uses filtered wavelengths to enhance the microvascular pattern and its alterations are associated with preneoplastic and neoplastic transformation of the upper aerodigestive tract mucosa (12–16). NBI using blue light (wavelength peak of 400 to 430 nm) and green light (wavelength peak of 515 to 555 nm), which correspond to the absorption peaks of hemoglobin (12), enhances the physicians’ chances to detect and delineate the suspicious mucosal lesions in a non-invasive way, and is thus beneficial to the diagnosis of a variety of benign and malignant lesions (14). NBI proved to be a useful diagnostic tool for the assessment of laryngeal leukoplakia; however, there is no clear place or recommendations for using NBI in preoperative assessments of VFL (17, 18) or establishing this method as the guidance for management (19)

The researchers’ hypothesis assumes the confirmation of a simple diagnostic schedule by using WL and NBI for patients with laryngeal leukoplakia in stratifying cancer risk to avoid false negative or false positive histological findings and vocal fold damage.

Therefore, the primary aim of this paper is to present the gathered and analyzed data to express which factors increase the risk of malignant transformation inside leukoplakia to avoid under- or overtreatment.

The second goal of our study was to confirm the usefulness of a clear diagnostic and decision-making schedule in which the timing and need for surgical intervention are crucial to not miss this cancer hidden underneath the white plaque.

## Material and Methods

### Study Population

A prospective study was conducted according to the guidelines of the Declaration of Helsinki and approved by the Ethics Committee of the Medical University of Lodz (RNN/225/19/KE, 9 April 2019) at the Poznan and Lodz Otolaryngology Tertiary Referral University Departments, between January 2015 and March 2020.

259 consecutive patients were enrolled – 212 men (81.85% of the cohort), 47 women (18.15%). The mean age was 62.08 years for males, and 61.2 years for females. VFL was diagnosed on 296 vocal folds.

The inclusion criteria were: a diagnosis of vocal fold leukoplakia confirmed by endoscopic evaluation under white light and NBI, no prior vocal fold-related medical intervention or procedures. Exclusion criteria were: other benign lesions (cysts, polyps, Reinke’s edema, or papilloma) in endoscopic evaluation; a history of laryngeal surgery, trauma, or intubation; a history of radiotherapy and chemotherapy for head and neck, as well as a lack of written consent from the patient and the presence of changes in endoscopic evaluation suggestive of an advanced neoplasm.

### Clinical Diagnostic Work-Up

All patients were assessed with a flexible transnasal video endoscope (Olympus Medical Systems Corporation, Tokyo, Japan) by means of WL and NBI. In the first step, the VFL in white light was observed regarding the texture, color, size, redness, symmetry, and thickness (according to the Chen 2019 classification). A fingertip control switch on the endoscope then changed the view to the NBI mode, and the vascular pattern was assessed, with close attention paid to the presence of intraepithelial papillary capillary loops (IPCLs) (according to the Ni 2019 (20) and European Laryngological Society (ELS) 2015 classifications).

Two independent physicians from each institution (JJ, HK, WP, JM), with at least 3 years of experience in the use of NBI, independently assessed each patient. For high-risk leukoplakia in the Chen classification, the cut-off point was 3 (elevated and rough leukoplakia); in the Ni classification, the cut-off point was 5 (IPCLs outside leukoplakia); and in the ELS classification, the cut-off point was 2 (perpendicular vessels).

Based on these classifications, the patients were qualified as at low or high risk of leukoplakia. The combination of these three classifications and cut-off points in the preoperative assessment of VFL was described by Pietruszewska et al. (2021) (9). Therefore, we used this scoring methodology for the leukoplakia image in WL and NBI.

In addition to the WL and NBI classification scores, additional variables were the patient’s age, sex, smoking habits, uni- or bilateral lesions, anterior commissure (AC) involvement, and the uni- or multifocal nature of the lesions.

All patients underwent transoral microsurgery by cold instrumentation, and specimens were sent for final histology. A frozen section was not performed due to the very small size of the specimens, which encompassed only the epithelial layer.

Histopathological diagnosis was performed according to the WHO classification system to classify the resected tissue as low-risk or high-risk dysplasia (21). The main predictive variables taken into consideration were the vascular pattern according to the Ni classification (2019), the ELS classification (2015), and the morphological characteristics according to the Chen classification (2019), along with the final histological findings.

### Statistical Methodology

**Figure 1:**
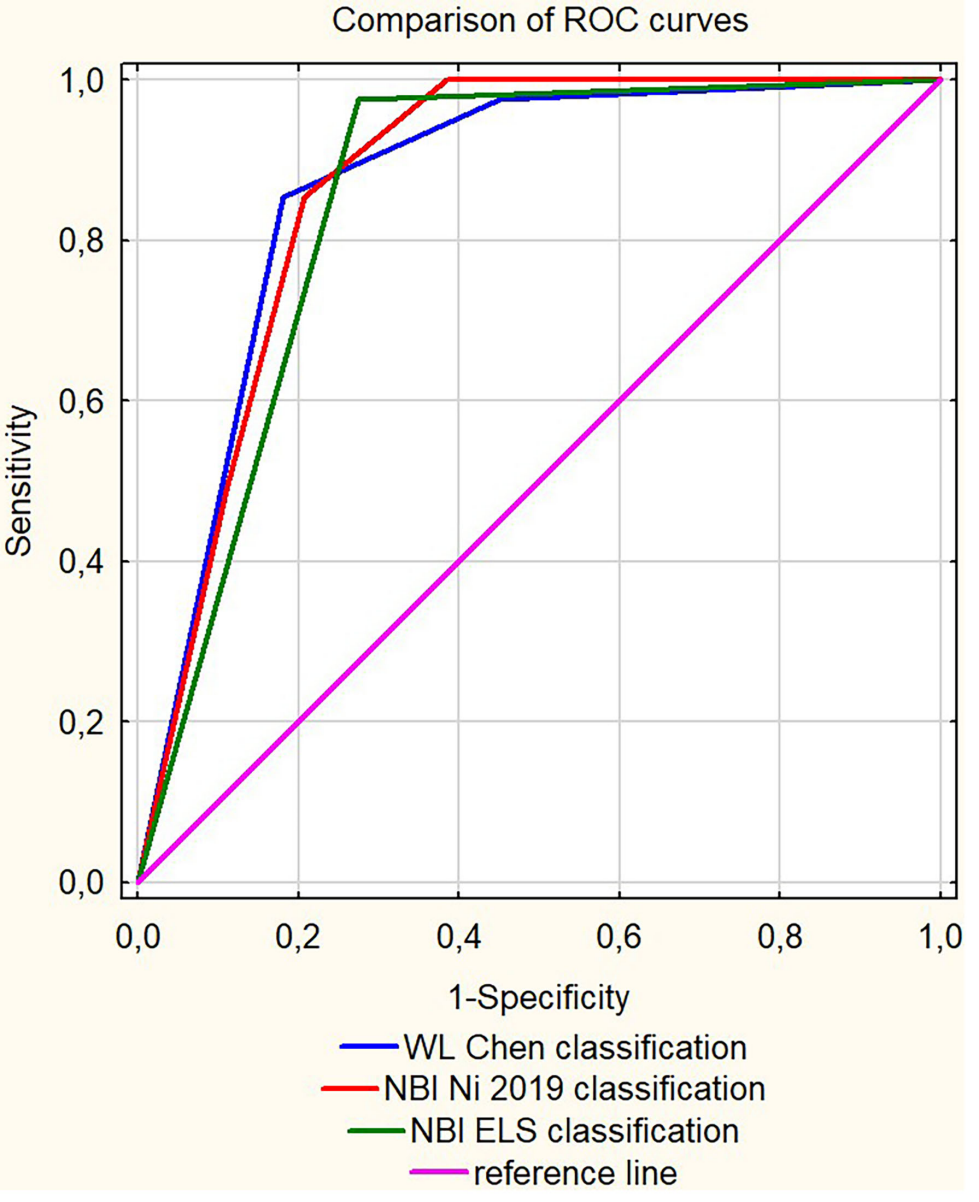
The receiver operating characteristic (ROC) curves comparison for the analyzed clinical classifications.

Data were stored in a computer-based filing system and reported as absolute and relative frequencies. Statistical analysis was performed in STATISTICA 13.1 Software (Dell, USA). The cut-off values for classifications with more than two degrees were established based on the receiver operating characteristic (ROC) curve analysis, and the highest Youden’s index in each classification determined the proposed cut-off value, as per Fluss et al. (22). To assess the diagnostic performance of the clinical classifications, the measures of occurrence (sensitivity, specificity, and accuracy) and the possibility of discriminating (positive and negative predictive values) for clinical classifications of WL and NBI endoscopy were calculated per the determined cut-off values. ROC curve comparisons for the analyzed classifications are presented in **Figure 1**. Odds Ratio’s for particular types of non-dychotomized classification systems by Ni 2019 and Chen 2019 are provided as **Supplementary Material** (**Supplementary Figures 1**, **2**).

#### Multivariate Analysis

Multivariate analysis was performed by means of logistic regression, the results of which are presented in a format suggested by Peng et al. (23) To perform logistic regression analysis on the probability of developing invasive cancer from leukoplakia, the following leukoplakia and epidemiological characteristics were analyzed in a univariate analysis: age, gender, leukoplakia localization, the focality of the leukoplakia, involvement of the anterior commissure, history of smoking, and the results of clinical classifications in WL and NBI. All variables were analyzed using the Likelihood Ratio (LR) test. The test was considered statistically significant if the *p*-value was < 0.05. The LR test results and the inclusion of variables for further multivariate analysis are summarised in **Table 1**.

**Table 1:** Summary of univariate analysis conducted as the first step towards formulating a logistic regression model.

Variable	P (LR) Univariate analysis	Included in further analysis	OR (95%CI)
**Age**	0.068	No	1.033 (0.997;1.071)
**Age categorized** 0: below 60. 1: 60 and older	0.017	Yes	2.53 (1.124;5.708)
**Gender** 0: male 1: female	0.196	No	1.844 (0.069;4.933)
**Leukoplakia localization** 0: unilateral 1: bilateral	<0.001	Yes	0.03 (0; 0.42)^#^
**Focality of leukoplakia** 0: unifocal 1: multifocal	0.877	No	0.49 (0.486;1.852)
**Anterior commissure involvement** 0: not involved 1: involved	0.049	Yes	1.91 (1.03;3.802)
**Smoking** 0: non-smoker 1: current or former smoker	0.014	Yes	3.646 (1.085;12.25)
**WL classification acc. to Chen’s 2019 classification** 0: stage I and II 1: stage III	<0.001	Yes	26.504 (10.531;66.705)
**NBI ELS classification** 0: longitudinal vessels 1: perpendicular vessels	<0.001	Yes	105.71 (14.259;783.725)
**NBI Ni’s 2019 classification** 0: stage to IV 1: stage V and VI	<0.001	Yes	22.23 (8.884;55.641)

Haldane-Anscombe correction was applied to account for cells with 0 cases.

The variables were checked for interactions and linearity of predictors using the LR test. No interactions between variables were detected. The linearity test result for age was *p* = 0.59; therefore, the variable was linear. A mixed effects logistic regression model was constructed to model a binary outcome of 0 (not developing invasive cancer) or 1 (developing invasive cancer under the leukoplakia). The equation to predict the probability of developing invasive cancer under the leukoplakia is presented below:


Predicted logit=(−7,753+(1,628)·Anterior Commisure Involvement+(2,496)·WL+(3,742)·ELS)

**Figure 2:**
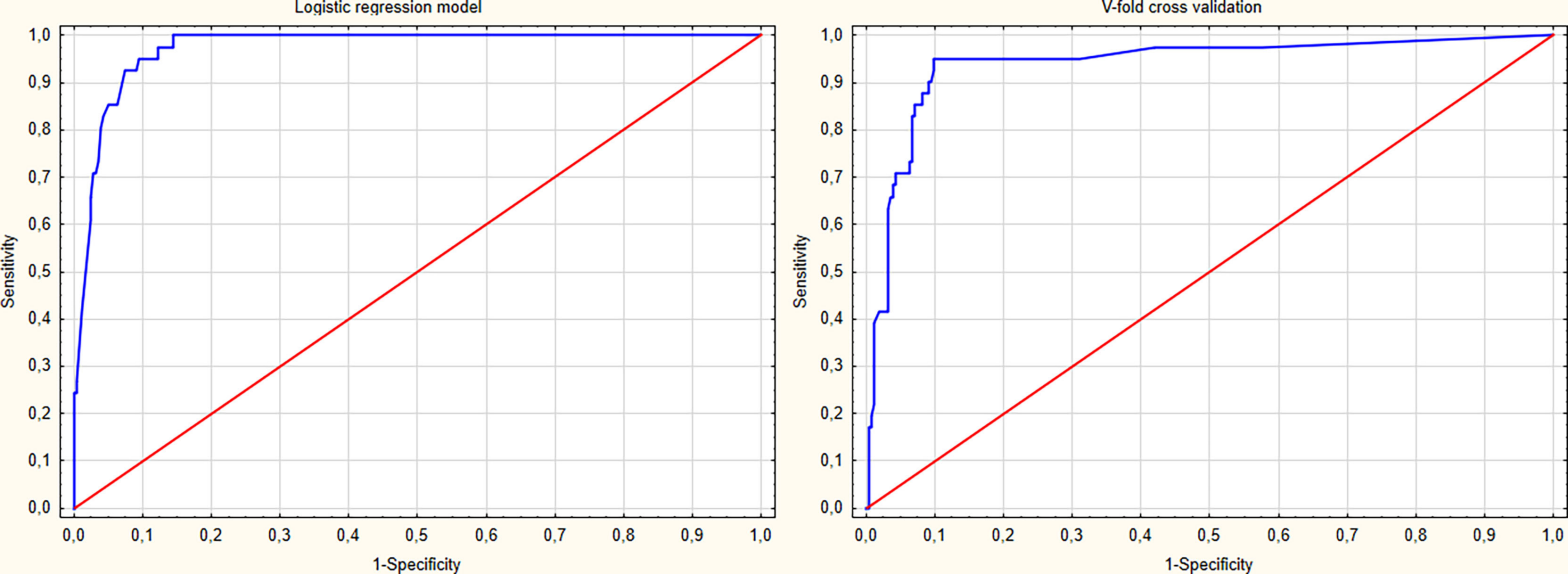
The receiver operating characteristic (ROC) curves of the proposed logistic regression model and the v-fold cross validation of the model.

The model’s predictive ability was validated using the V-fold cross-validation method with 10 subsets of data. The model’s goodness of fit was evaluated with the Hosmer-Lemeshow test (p=0.623). The discrimination curves for the regression model and the V-fold cross-validation are depicted in **Figure 2**. The results of the multivariate analysis are summarised in **Table 2**. The predictive and diagnostic capabilities of the model were evaluated using ROC analysis, which is shown in **Table 3**.

**Table 2:** Summary of multivariate analysis using logistic regression, including the parameters for the regression model and goodness of fit test results.

Predictor	β	*SE*β	Wald’s χ^2^	d*f*	p	e^β^ (Odds Ratio) [95%CI]
**Constant**	-7.753	3.373	5.285	1	0.022	NA
**Anterior commissure involvement** 0: not involved 1: involved	1.628	0.684	5.672	1	0.017	5.094 [1.334;19.452]
**WL Chen’s 2019 classification** 0: stage I and II 1: stage III	2.496	0.687	13.203	1	<0.001	12.134 (3.157;46.638)
**NBI ELS classification** 0: longitudinal vessels 1: perpendicular vessels	3.742	1.144	10.702	1	0.001	42.183 (4.482;397.015)

NA, not applicable.

**Table 3:** Diagnostic performance of the analyzed endoscopic classifications and proposed cut-off values for determining the risk of cancer development under vocal fold leukoplakia.

Clinical classification	Youden’s index	Proposed cut-off value	AUC	Sensitivity	Specificity	PPV	NPV
WL Chen’s 2019 classification	0.67	3	0.867	85.4%	82%	43.2%	97.2%
Narrow Band Imaging ELS classification	0.7	1	0.851	97.6%	72.5%	36.4%	99.5%
NBI Ni’s 2019 classification	0.65	5	0.823	85.4%	79.2%	39.8%	97.1%
Proposed logistic regression model			0.973	92.7%	92.9%	67.9%	98.8%

AUC, Area under curve; PPV, positive predictive value; NPV, negative predictive value; WL, white light; NBI, narrow band imaging

## Results

Invasive cancer was confirmed in 41 (13.6%) out of the 296 VFL specimens in the final histology. Among this group, the mean age was 64.24, with 5 (12.2%) females and 36 (87.8%) males. 38 (92.7%) of the 41 patients were heavy smokers, and 23 (56.1%) were heavy drinkers. Taking into consideration the precise localization of the plaque carrying cancer, all 41 (100%) specimens were unilateral, but in 16 (39%), the lesions spread in the AC. Taking into account the number of VFL points, 24 (58.5%) presented unifocal and 17 (41.5%) multifocal. The complete characteristics of the study group divided by histopathological results Are available in the **Supplementary Material**.

**Figure 3:**
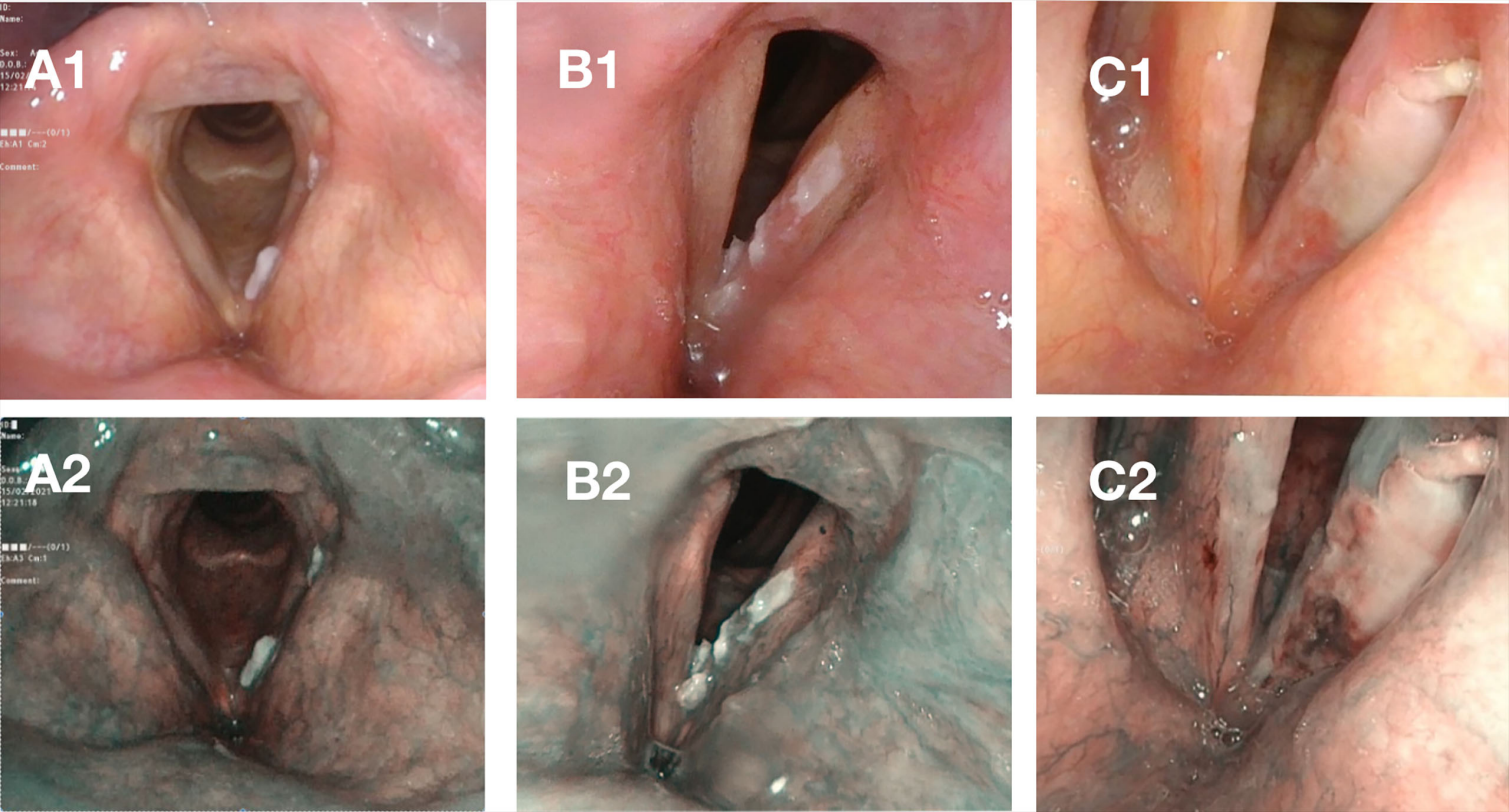
Case A – elevated and smooth leukoplakia seen in white light [WL] **(A1)** and Narrow Band Imaging [NBI] **(A2)** with no evidence of neoplastic proliferation and only low-grade dysplasia in histopathological examination. Case B – elevated and rough leukoplakia in WL **(B1)** with pathologic vessels visible in the periphery of the plaque in NBI **(B2)**, high-grade dysplasia in histopathological examination. Case C – elevated and rough leukoplakia **(C1)** with pathologic vessels visible in the periphery of leukoplakia on the frontal part of the left vocal fold in NBI **(C2)** with invasive carcinoma in histopathological examination.

**Figure 3**. Leukoplakia in WL (acc. to Chen type II) and NBI (acc. to Ni type V, acc. to ELS type II).

Using the Chen classification, out of the total number of 41 lesions only 1 (2.4%) was assessed as type 1 (flat and smooth), 5 (12.2%) as type 2 (elevated and smooth), and 35 (85.4%) as type 3 (rough).

Using the Ni classification, 2 (4.9%) lesions were assessed as type 3 (IPCLs can be seen at the surface of the vocal fold mucosa surrounding the leukoplakia without clear boundaries), 4 (9.8%) as type 4 (IPCLs can be observed on the surface of the white plaque), 15 (36.6%) as type 5 (IPCLs can be seen on the vocal fold mucosa outside the leukoplakia with regular boundaries), and 20 (48.8%) as type 6 (IPCLs can be seen on the vocal fold mucosa outside the leukoplakia and on the surface of the plaque).

Using the ELS classification: 1 (0.4%) of the 41 had type 1 (longitudinal vascular pattern) and 40 (97.5%) had type 2 (perpendicular vascular pattern) leukoplakia.

### Univariate Analysis

Univariate analysis revealed that bilateral vocal fold changes were 0.03 times less likely to present as invasive cancer (*p* < 0.001). However, this observation was not included in the regression model because there were no cases of invasive cancer present in bilateral VFL.

Considering age as a continuous variable, the LR test results were close to statistical significance (p=0.068). However, in order to include age in the multivariate analysis, age was dichotomized using the Weight of Effect (WOE), and similar WOE values were combined into two categories: patients up to 60 years and older than 60. In this case, people over 60 were 2.53 times more likely to develop cancer under the leukoplakia (p=0.017). A history of smoking (present or former tobacco users) was associated with a 3.646 times increase in the probability of developing cancer. Neither gender nor the focality of the leukoplakia (multifocal or unifocal plaques on one vocal fold) was associated with statistically significant odds of malignant transformation under VFL.

### Multivariate Analysis

In the multivariate analysis using a logistic regression model, VFL involving the anterior commissure was 5.094 times more likely to present with invasive cancer (p=0.017). The plaques that were rough and elevated (stage III in WL) were also associated with significantly higher odds of developing cancer (OR 12.124, p <0.001). The presence of a perpendicular blood vessel pattern surrounding the plaque (ELS grade 2) was associated with a 42.183 times higher risk of malignant transformation (p <0.001). The results of the multivariate analysis are presented in **Table 2**.

### Diagnostic Performance

To evaluate the diagnostic performance of different clinical classifications regarding the risk of malignant transformation in clinical leukoplakia, we compared the AUC (Area Under Curve) values, sensitivity, specificity, and positive and negative predictive values. Among the clinical classifications proposed by different authors, the highest AUC of 0.867 in the ROC analysis was reported for WL evaluation according to Chen et al. (2019), with a sensitivity of 85.4% and specificity of 82%, and Negative Predictive Value (NPV) at 97.2%; however, Positive Predictive Value (PPV) was low at 43.2%.

The evaluation of leukoplakia with NBI utilizing the Ni 2019 classification did not significantly improve the diagnostic performance, with specificity being lower than WL (79.2% *vs.* 82%), sensitivity at the same level, similar to NPV of 97.1%, and an even lower PPV (39.8% *vs.* 43.2%).

Introducing the ELS classification to the NBI endoscopy shows better results than the Ni 2019 classification, with slightly higher AUC at 0.851, higher sensitivity (97.6% *vs.* 85.4%) but with lower specificity (72.5% *vs.* 79.2%).

The diagnostic algorithm derived from the logistic regression analysis, which included evaluation of a patient in NBI, WL, and evaluation of anterior commissure involvement, improves the clinical utility of these classifications and shows improved diagnostic performance. The AUC of this model was the highest (0.973), with the highest sensitivity, specificity, PPV, and NPV (90.2%, 89%, 56.9%, and 98.3%, respectively).

## Discussion

In the presented study, we have shown the tactics to resolve the clinical challenges that laryngologists face balancing therapeutic decisions in vocal fold leukoplakia. The philosophy of managing such patients is to consider both oncological efficacy and functional outcomes. Thus, our hypothesis assumed that a combination of procedures (WL and NBI), scoring systems (combining multiple image classifications in WL and NBI), patient-dependent variables, and the precise location of the plaque contribute to the ability to not miss the cancer.

Since its first introduction in the late 1990s, the use of NBI has considerably contributed to physicians’ ability to non-invasively detect and delineate the suspicious mucosal lesions (19). Nowadays, NBI is a mainstay of diagnostics, although in VFL, it has some limitations connected with the umbrella effect due to thick layers of keratin covering the vascular pattern. Nevertheless, in recent years, this impediment has been overcome through the assessment of the vascularization outside the plaque, and a number of studies have proved that NBI can be applied with success in VFL (24–26). But the problem remains in standardizing cut-off points and translating the NBI findings into firm indications for surgery or, on the contrary, to continue with watchful waiting.

Different classifications in recent decades (12, 16–19) were proposed. However, there is still the need for a common, unified schedule to be shared among clinicians to describe WL findings and NBI-enhanced vascular patterns to distinguish the nature of VFL.

In 2015, ELS introduced a simplified classification for vascular changes of the vocal folds divided between longitudinal and perpendicular vascular changes. The perpendicular changes present as intraepithelial papillary capillary loops (IPCLs) that are connected with laryngeal papillomatosis, precancerous, and cancerous lesions (27). Many authors confirmed the high accuracy of ELS classification in determining between benign and malignant lesions (28–30). In 2019, Ni et al. improved the classification from 2011, adding another six types that cover vocal fold leukoplakia. Both classifications are based on the morphological changes present in laryngeal IPCLs to distinguish them between benign and malignant lesions (31). In the new classification, the focality is on the presence of perpendicular vessels outside or on the surface of the plaque, which suggests a malignant lesion. The pathological vessels seen in NBI are visualized as brown dots of different sizes and are twisted, earthworm-like. The accuracy of this classification in the assessment of VFL was 90.8%, which is significantly better than that of conventional WL endoscopy (70%) (31, 32).

Our paper presented similar results. Using NBI in laryngeal leukoplakia diagnosis revealed high accuracy, especially when using the ELS classification. Specifically, the accuracy was 93.9%, according to ELS, and 85.1%, according to the NI 2019 classification. The combined use of contact endoscopy (CE) and NBI has already been suggested for visualizing specific vascular changes indicative of glottic neoplasia (33). However, inter-rater reliability and agreement in three classification systems proved to be the best for vascular changes by the ELS and was significantly higher than those by Ni et al. and Puxeddu et al. (33).

Other than NBI endoscopy, systems like the Storz Professional Image Enhancement System have also been increasingly used in patients with suspected lesions of the larynx and hypopharynx (34). Both methods are comparable in the detection and analysis of superficial neoangiogenesis, and both methods are efficient in observing epithelial and subepithelial microvascular irregularities and pathologies, but NBI has been more popularized (35). A conventional laryngoscope using white light plays the main role in the decision-making process concerning VFL in the majority of laryngology departments. In 2019, Chen et al. proposed a new WL classification for VFL connected with the morphological features of the lesions, and distinguished three types of plaque: flat and smooth, elevated and smooth, and rough. They showed a high correlation between the morphological features of the laryngeal leukoplakia and their final histology result (19).

Another issue is the precise location of the lesion in the glottis area. It is known that the anterior commissure raises oncological concern because it represents a weak point with regard to tumor spread (36, 37). There are different degrees of AC involvement (36), but this stratification is concerned with tumor infiltration and not with the location of the leukoplakia. The very small distance between anterior commissure mucosa and thyroid cartilage, and the lack of perichondrium or periosteum in the AC area promote the spread of cancer, even in early invasion (38, 39). This should not affect the risk of cancer in VFL, which affects the superficial epithelial layers. Nevertheless, the AC is a site susceptible to the influence of tobacco smoke carcinogens on the epithelium.

Thus, we wanted to check whether the plaque in the AC location should be treated more suspiciously. We confirmed that this area should receive greater attention because the odds of cancer developing under a leukoplakia plaque in lesions involving AC were 5.094 times higher than those not involving the anterior commissure.

In this paper, we stabilize the schedule to recognize cancer under VFL plaque on a large, multicenter group of patients. The flowchart used in this research followed the schedule published by Pietruszewska et al., but we add an additional variable, AC involvement, which in our opinion, is crucial for cancer prediction. The algorithm includes a scheme of action based on a physical examination: morphology of plaque in WL according to the Chen classification, the vascular pattern in NBI scored according to the ELS classification, and the precise localization of the plaque.

Our results showed a high correlation between both the ELS and Chen classifications and the pathological outcomes, which is comparable to the results of Lin et al. (40) and Lu et al. (41). Therefore, we believe that the combined classifications of ELS and Chen, and the addition of the focus on AC involvement play a basic role in the diagnosis of laryngeal leukoplakia, especially in determining and distinguishing between those that are benign and those that are malignant. These findings have clinical importance for initial VFL diagnosis, directing patients for surgery, and routine endoscopic surveillance.

This study has some strengths and limitations. The strong points of this study include the creation of a new schedule for VFL diagnostics based on WL and NBI with regard to AC location in one of the biggest clinical groups of VFL. The combination of methods applied according to the proposed schedule proved effective in distinguishing cancer underneath the leukoplakia and gives direct suggestions for treatment options. However, there are still several weaknesses in the study. The main issue concerns the prevalence of NBI in laryngology departments; this method is not as popular as white light endoscopy, and additionally, the learning curve of NBI is quite long.

## Conclusions

The ability to detect invasive cancer under leukoplakia remains a diagnostic challenge. An algorithm that combines WL and NBI, and the combination of two classifications, improves the specificity and positive predictive value, especially in anterior commissure involvement.

## Data Availability Statement

The raw data supporting the conclusions of this article will be made available by the authors, without undue reservation.

## Ethics Statement

The studies involving human participants were reviewed and approved by Komisja Bioetyczna przy Uniwersytecie Medycznym im. Karola Marcinkowskiego w Poznaniu/Bioethics Committee of Poznań University of Medical Sciences/. Written informed consent for participation was not required for this study in accordance with the national legislation and the institutional requirements.

## Author Contributions

MW and WP contributed to conception and design of the study. HK, PN, OR, and JM organized the database. OR performed the statistical analysis. HK wrote the first draft of the manuscript. HK, OR, MW, and WP wrote sections of the manuscript. All authors contributed to manuscript revision, read, and approved the submitted version.

## Conflict of Interest

The authors declare that the research was conducted in the absence of any commercial or financial relationships that could be construed as a potential conflict of interest.

## Publisher’s Note

All claims expressed in this article are solely those of the authors and do not necessarily represent those of their affiliated organizations, or those of the publisher, the editors and the reviewers. Any product that may be evaluated in this article, or claim that may be made by its manufacturer, is not guaranteed or endorsed by the publisher.
