# Supplementary figures and images for: Leukoplakia: An Invasive Cancer Hidden within the Vocal Folds. A Multivariate Analysis of Risk Factors
Source: Front Oncol. 2021 Dec 13;11:772255. doi: 10.3389/fonc.2021.772255 (PMC8711120; doi:10.3389/fonc.2021.772255)

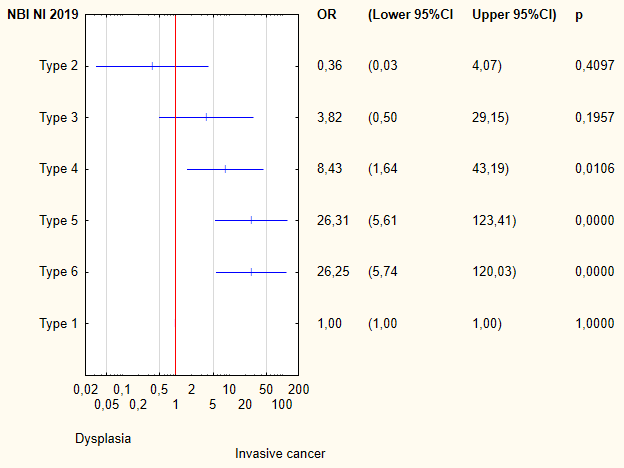

Supplement: Supplementary file 1 [file Image_1.png]

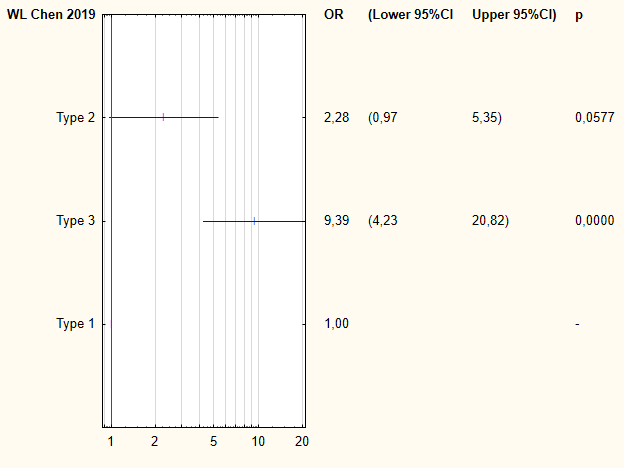

Supplement: Supplementary file 2 [file Image_2.png]
