# Supplementary material for: Leukoplakia: An Invasive Cancer Hidden within the Vocal Folds. A Multivariate Analysis of Risk Factors
Source: Front Oncol. 2021 Dec 13;11:772255. doi: 10.3389/fonc.2021.772255 (PMC8711120; doi:10.3389/fonc.2021.772255)
Supplement: Supplementary file 3 [file Table_1.docx]

Supplementary Material

| Variable | Low-grade dysplasia | High-grade dysplasia | Invasive cancer | Sum by row |
| --- | --- | --- | --- | --- |
| Male | 149 | 54 | 36 | 239 |
| Female | 38 | 14 | 5 | 57 |
| Unilateral VFL | 129 | 45 | 41 | 215 |
| Bilateral VFL | 58 | 23 | 0 | 81 |
| Unifocal VFL | 109 | 37 | 24 | 170 |
| Multifocal VFL | 78 | 31 | 17 | 126 |
| AC not involved | 148 | 43 | 25 | 216 |
| AC involved | 39 | 25 | 16 | 80 |
| WL stage I | 121 | 19 | 1 | 141 |
| WL stage II | 51 | 18 | 5 | 74 |
| WL stage III | 15 | 31 | 35 | 81 |
| ELS grade 1 | 157 | 28 | 1 | 186 |
| ELS grade 2 | 30 | 40 | 40 | 110 |
| Ni 2019 stage I | 64 | 2 | 0 | 66 |
| Ni 2019 stage II | 74 | 17 | 0 | 91 |
| Ni 2019 stage III | 14 | 1 | 2 | 17 |
| Ni 2019 stage IV | 22 | 8 | 4 | 34 |
| Ni 2019 stage V | 6 | 19 | 15 | 40 |
| Ni 2019 stage VI | 7 | 21 | 20 | 48 |
| Non-smoker | 42 | 15 | 3 | 60 |
| Current and former smoker | 145 | 53 | 38 | 236 |
| Below 60 y.o. | 70 | 27 | 8 | 105 |
| 60 y.o and above | 117 | 41 | 33 | 191 |
| Total cases of VFL | **187** | **68** | **41** | **296** |

VFL – vocal fold leukoplakia, AC – anterior commissure, ELS – European laryngological society, y.o – years old.
